# A Novel, Non-canonical Splice Variant of the *Ikaros* Gene Is Aberrantly Expressed in B-cell Lymphoproliferative Disorders

**DOI:** 10.1371/journal.pone.0068080

**Published:** 2013-07-09

**Authors:** Daria Capece, Francesca Zazzeroni, Maria Michela Mancarelli, Daniela Verzella, Mariafausta Fischietti, Ambra Di Tommaso, Rita Maccarone, Sara Plebani, Mauro Di Ianni, Alberto Gulino, Edoardo Alesse

**Affiliations:** 1 Department of Biotechnological and Applied Clinical Sciences, University of L’Aquila, L’Aquila, Italy; 2 Department of Life, Health and Environmental Sciences, University of L’Aquila, L’Aquila, Italy; 3 Department of Molecular Medicine, “Sapienza” University of Rome, Rome, Italy; Kanazawa University, Japan

## Abstract

The *Ikaros* gene encodes a Krüppel-like zinc-finger transcription factor involved in hematopoiesis regulation. Ikaros has been established as one of the most clinically relevant tumor suppressors in several hematological malignancies. In fact, expression of dominant negative Ikaros isoforms is associated with adult B-cell acute lymphoblastic leukemia, myelodysplastic syndrome, acute myeloid leukemia and adult and juvenile chronic myeloid leukemia. Here, we report the isolation of a novel, non-canonical Ikaros splice variant, called Ikaros 11 (Ik11). Ik11 is structurally related to known dominant negative Ikaros isoforms, due to the lack of a functional DNA-binding domain. Interestingly, Ik11 is the first Ikaros splice variant missing the transcriptional activation domain. Indeed, we demonstrated that Ik11 works as a dominant negative protein, being able to dimerize with Ikaros DNA-binding isoforms and inhibit their functions, at least in part by retaining them in the cytoplasm. Notably, we demonstrated that Ik11 is the first dominant negative Ikaros isoform to be aberrantly expressed in B-cell lymphoproliferative disorders, such as chronic lymphocytic leukemia. Aberrant expression of Ik11 interferes with both proliferation and apoptotic pathways, providing a mechanism for Ik11 involvement in tumor pathogenesis. Thus, Ik11 could represent a novel marker for B-cell lymphoproliferative disorders.

## Introduction

Ikaros is a member of the Krüppel-like zinc finger transcription factor family. It is encoded by the *IKZF1* gene, which consists of eight exons alternatively spliced to produce different isoforms able to homo- and heterodimerize [1]–[3]. All isoforms share two C-terminal zinc-finger domains that allow for homo- and heterodimerization between Ikaros family members, while they differ in the number of N-terminal zinc-finger motifs, which form the DNA-binding domain. Ikaros proteins with fewer than three N-terminal zinc-fingers act as dominant negative (DN) factors, being able to impair the activity of the DNA-binding isoforms [1], [4].

Ikaros has been shown to act both as a canonical transcriptional activator and repressor. In addition, a large body of literature has demonstrated that it may modulate gene expression by taking part in chromatin remodeling [1]–[3], [5].

Ikaros is a master regulator of hematopoiesis, especially of lymphoid development. Initial studies in *Ikaros* null mice showed an early and complete block of B-cell development, the absence of natural killer and dendritic cells, as well as a decreased number of T cells and perturbed myelopoiesis [6], [7]. In addition, mice lacking Ikaros or expressing DN isoforms developed T-cell leukemia, suggesting that Ikaros acts as a tumor suppressor gene in the lymphoid lineage [8].

Ikaros’ role in tumor suppression is due to its ability to negatively regulate the G_1_/S transition through the modulation of both positive and negative effectors of the cell cycle [9]–[11]. Ikaros is also involved in apoptosis. *Ikaros* null mice showed decreased apoptosis in response to oxidative stress in bone marrow erythroid cells [12]. In addition, the overexpression of full-length Ikaros increased apoptosis in leukemic cell lines [13].

Genetic inactivation of *Ikaros* and the aberrant expression of DN isoforms have been demonstrated in different types of human leukemia, such as B and T acute lymphoblastic leukemia (ALL) [14]–[27], chronic myeloid leukemia (CML) [15], [28], [29] and acute myeloid leukemia (AML) [4]. Mechanisms underlying the generation of DN isoforms in tumors are still debated, but intragenic deletions were shown to be involved [15].

We identified a novel, non-canonical splice variant of the *Ikaros* gene, which we called *Ik11*. Here, we showed that Ik11 is a novel DN isoform capable of inactivating functional Ikaros isoforms by, at least in part, cytoplasmic sequestration. Ik11 promoted cell proliferation and counteracted apoptosis, *in vitro*. Finally, in order to investigate a possible involvement of Ik11 in tumor pathogenesis, we have also analyzed Ik11 expression in human CML, ALL, myelodysplastic syndromes, lymphomas and chronic lymphocytic leukemia (CLL). Interestingly, we found that Ik11 was aberrantly expressed particularly in B-cell lymphoproliferative disorders. To our knowledge, this is the first evidence for DN Ikaros expression in CLL.

## Materials and Methods

### Ethics Statement

This study was reviewed and approved by the Institutional Review Board of Molecular and Clinical Pathology and Oncology Section, Department of Biotechnological and Applied Clinical Sciences, University of L’Aquila (L’Aquila, Italy) in accordance with Declaration of Helsinki. Blood samples were collected with the written informed consent of the donors.

### IK11 Cloning

*Ikaros11* was isolated from Ficoll-separated normal human peripheral blood lymphocytes (hPBLs) using the SMART RACE kit (BD Biosciences, San Jose, CA, USA). 1 µg of RNA was subjected to reverse transcription PCR (RT-PCR) and subsequently amplified according to the manufacturer’s specifications. PCR products were cloned into the pcRII vector (TOPO-TA cloning, Life Technology, Carlsbad, CA, USA) and sequenced.

### Plasmids and Reagents

pcDNA3.1-Ik2, pcDNA3.1-Ik6, pcDNA3.1-Ik11, pcDNA3.1/Myc-HysB-Ik2, pcDNA3.1/Myc-HysB-Ik6 and pcDNA3.1/Myc-HysB-Ik11 constructs are described in Supporting Text S1. The pGL3 luciferase reporter vector containing an *Ikaros* responsive gene was previously described [30]. Staurosporine was purchased from Sigma Aldrich (St. Louis, Missouri USA). zVAD-FMK was purchased from Promega (Madison, WI, USA).

### Tumor Samples

cDNAs from patients with poorly differentiated malignant lymphoma (n = 1), Hodgkin’s lymphoma (n = 1) and non-Hodgkin’s lymphoma (n = 1) were purchased from BD Biosciences. Samples from patients with CML (n = 21), ALL (n = 11), myelodysplastic syndromes (n = 7), Hodgkin’s and non-Hodgkin’s lymphoma (n = 6) and CLL (n = 22) were provided from the Internal Medicine and Hematology Unit of San Salvatore Hospital-L’Aquila. Diagnosis was based on morphology, cytogenetics, immunophenotype and molecular biology analyses.

### Cell Lines and Transfection

293T HEK, Cos7 and RAW 264 (ATCC, Manassas, VA, USA) cell lines were cultured in Dulbecco’s Modified Eagle’s Medium supplemented with 10% heat-inactivated fetal bovine serum (FBS), 1% L-glutamine and 1% Penicillin Streptomycin. K562 (ATCC) and BJAB [31] cell lines were cultured in RPMI 1640 with 10% FBS, 1% L-glutamine and 1% Penicillin Streptomycin. PBLs were obtained by Ficoll density-gradient centrifugation of heparinized blood from healthy donors. Leukocyte subset isolation was performed by magnetic cell sorting (MACS, Miltenyi Biotec, Auburn, CA, USA) according to the manufacturer’s instructions. CD14 or CD19 mAb-coated microbeads and CD4 or CD8 cell isolation kits (Miltenyi Biotec) were used to purify monocytes, B and T cells, respectively.

The transfections were performed using Lipofectamine 2000 Reagent (Life Technology), Fugene HD (Promega) and Attractene Transfection Reagent (Qiagen, Valencia, CA, USA) according to the manufacturer’s specifications. K562 and BJAB were electroporated by using Amaxa-Nucleofector II™ according to the manufacturer’s specifications (Lonza, Walkersville, MD, USA).

### RNA Extraction, PCR and Real-time PCR

RNA was extracted with Trizol reagent (Life Technology) according to the manufacturer’s specifications and reverse transcribed using the GeneAmp® Gold RNA PCR Reagent Kit (Life Technology). cDNAs of spleen, thymus, lymph nodes and bone marrow were commercially available (BD Biosciences). Semiquantitative PCR was performed using Go-Taq Green Master Mix (Promega). Real-time PCR was performed by using Mastercycler® ep realplex and RealMasterMix Sybr Rox (Eppendorf, Barkhausen, Hamburg, Germany). The *gapdh* gene was used as a normalizing control. Primer sequences and Real-time PCR conditions are available upon request.

### Sequence Analysis

PCR products were sequenced using the ABI PRISM Big Dye terminator v3.1 cycle sequencing kit and the automated sequencer ABI PRISM 310 Genetic Analyzer (Life Technology) in accordance with the manufacturer’s instructions.

### *In vitro* Binding Assay, Immunoprecipitation and Western Blotting

*In vitro* translation of the Ikaros 2-myc protein was performed using the TNT Quick Coupled Transcription/Translation System (Promega), according to manufacturer’s instructions. pcDNA3.1/Myc-HysB-Ik2 construct was used as template. *In vitro* translation of the Ikaros 6 and Ikaros 11 proteins was performed using the Transcend Non-Radioactive Translation Detection System (Promega), according to manufacturer’s instructions. pcDNA3.1-Ik6 and pcDNA3.1-Ik11 were used as template. Translated proteins were incubate at 37° for 1 hour. IP buffer (20 mM Tris-HCl pH 7.4, 140 mM NaCl, 10% glycerol, 1 mM CaCl_2_, 0.1% Triton X, 1 tablet of complete mini EDTA-free protease inhibitors (Roche Molecular Biochemicals, Mannheim, Germany) and anti-Myc antibody (Santa Cruz Biotechnology, Inc, Santa Cruz, CA, USA) were subsequently added to the protein mix. After 1 hour incubation at RT on a rotating wheel, Protein A/G Plus-Agarose (Santa Cruz Biotechnology) was added to each protein mix and incubated at 4°C overnight.

Cell extracts were prepared in Giordano buffer (50 mM Tris-HCl pH 7.4, 250 mM NaCl, 5 mM EDTA, 25 mM NaF, 0.1% Triton X-100, 0.1 mM PMSF, 1 µg/ml Leupeptine, 10 µg/ml trypsin inhibitor from soybean, 10 µg/ml TPCK, 5 µg/ml TLCK, 1 µg/ml Aprotinine, 0.1 mM Na_3_VO_4_) or RIPA buffer (1x phosphate buffered saline, 1% NP40, 0.5% sodium deoxycholate, 1% SDS, 0.1 mM PMSF, 1 µg/ml aprotinine, 0.1 M Na_3_VO_4_) containing complete mini EDTA-free protease inhibitors (Roche Molecular Biochemicals). Immunoprecipitation was performed using anti-Myc antibody and Protein A/G Plus-Agarose (Santa Cruz Biotechnology), according to the manufacturer’s specifications. Primary antibodies were: Ikaros, Cyclin E, BAX, Actin (Santa Cruz Biotechnology), p27 (BD Biosciences), p21 and PARP (Cell Signaling Technologies, Inc Beverly, MA, USA).

### Luciferase Assay

The Ikaros-regulated promoter of the *KCTD11*(*REN*) gene was cloned into the pGL3 luciferase reporter vector as previously described [30]. Promoter activity was analyzed using the Dual-Luciferase Reporter Assay System (Promega) according to the manufacturer’s instructions. Promoter activity was calculated as the ratio of firefly luciferase activity to Renilla luciferase activity.

### Immunofluorescence Assay

Transfected cells were cultured on poly-D-Lysine, 8 wells culture slides (BD) and fixed in 4% paraformaldehyde. Cells were incubated with anti-Myc and/or anti-Ikaros antibodies (Santa Cruz Biotechnology) followed by Fluorescein (FITC)-conjugated AffiniPure Goat anti-Rabbit IgG (H+L) and Texas Red dye conjugated AffiniPure Goat anti-mouse IgG (H+L) secondary antibody (Jackson ImmunoResearch, West Grove, PA, USA). Nuclei were stained with Hoechst 33258 (Life Technology). The subcellular localizations were analyzed using a Leica TCS SP5 Confocal Microscopy. 40x objective was used.

### MTS Assay

CellTiter 96® Aq_ueous_ One Solution Reagent (Promega) was added to transfected cells according to the manufacturer’s instructions. Cell viability was determined by measuring the absorbance at 490 nm using a µ-Quant plate-reader (Bio-Tek Instruments, Winooski, VT, USA) and calculated as the percent of control (pcDNA3.1). Statistical analysis was performed using unpaired 2-tailed Student’s *t* test. *P* values less than 0.05 were considered significant.

### Apoptosis Evaluation

Caspase-3 activity was measured by using the CaspACE™ Assay System, Colorimetric (Promega), according to the manufacturer’s instructions and calculated as the relative absorbance (mean induced apoptosis sample A_405_ -mean negative control sample A_405_). Measurement of mono- and oligonucleosome enrichment was carried out by ELISA (Cell Death Detection ELISA, Roche Molecular Biochemicals), according to the manufacturer’s instructions. The mono and oligonucleosome enrichment was calculated as the ratio of mean induced apoptosis sample A_405_ and mean negative control sample A_405_. Statistical analysis was performed using the unpaired 2-tailed Student’s *t* test. *P* values less than 0.05 were considered significant.

## Results

### Ik-11: A Novel, Non-canonical Splice Variant of Ikaros

**Figure 1:**
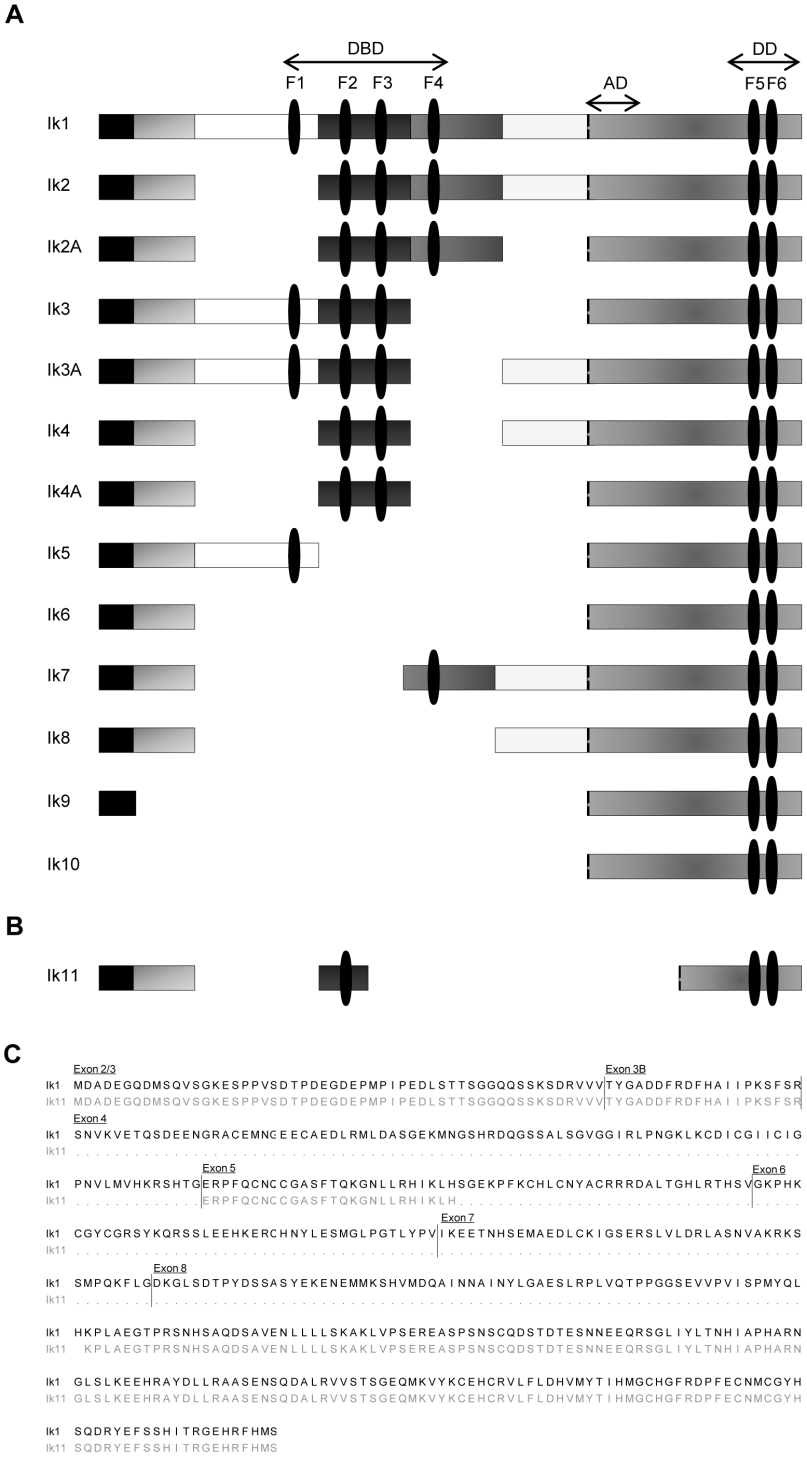
Ikaros 11 is a novel, non-canonical splice variant of the *Ikaros* gene. (**A**) Diagrammatic representation of *Ikaros* isoforms 1–10 with their functional domains. (**B**) Schematic representation of the novel splice variant Ik11. (DBD, *DNA Binding Domain*; AD, *Activation Domain*; DD, *Dimerization Domain*, F1–F6, *Zinc Finger modules*). (**C**) Amino acid sequence alignment of the full-length Ik1 and Ik11. Exon positions are indicated. Exon 3B has been previously described [16], [25], [36] but is currently not identified as an exon in Gene Bank.

Several isoforms of the *Ikaros* gene have been previously identified (Figure 1A) [1]–[3], [15], [16], [23]–[25], [32]–[35], as either full-length proteins with transcriptional activity or splice variants with dominant-negative functions. Due to the relevance of these latter forms in the development of hematological malignancies, we decided to perform a PCR-based screening to identify novel *Ikaros* splice variant mRNAs. To this end, mRNA from hPBLs was reverse transcribed and cDNA was amplified by using the SMART RACE kit. We isolated several isoforms of *Ikaros*, which have been sub-cloned and sequenced. Among these, one was unknown and it was called *Ik11* (GeneBank Acc. N. JX459579). *Ik11* is a non-canonical splice variant, as revealed by DNA sequencing. The *Ik11* sequence contains exon 2, exon 3, the first half of exon 5 (from the 1^st^ amino acid [E] to the 26^th^ amino acid [H]) and the second half of exon 8 (from the 68^th^ amino acid [K] to the last amino acid [S]) (Figure 1B–C). Thus, the Ik11 protein presents two C-terminal and only one N-terminal zinc-finger domains, whereas it completely lacks the transcriptional activation domain (AD) (Figure 1A–B and Figure S1), suggesting that Ik11 might dimerize with other Ikaros isoforms but not induce gene transcription. In addition, Ik11 had the 60-bp insertion at the end of exon 3 that was already described in other Ikaros isoforms [16], [25] and referred to as exon 3B [36].

**Figure 2:**
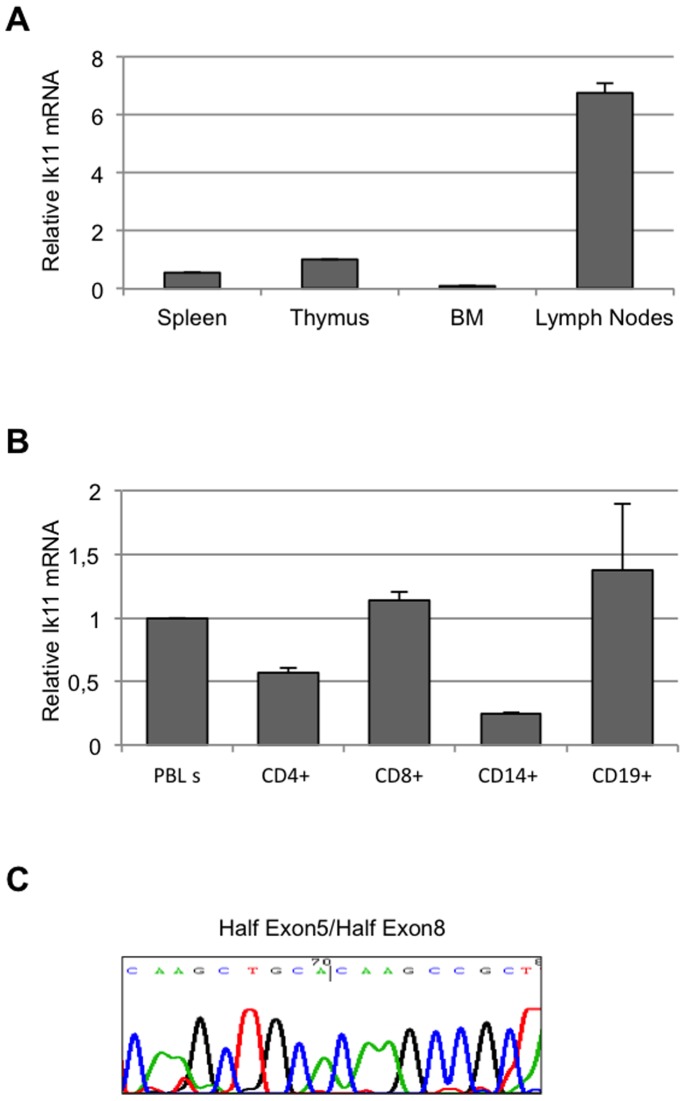
Ik11 expression is restricted to lymph nodes and peripheral lymphocytes. (**A**) Real-time PCR analysis of *Ikaros* mRNA in human cDNAs from normal thymus, spleen, lymph nodes and bone marrow. The Ik11 levels are expressed as fold change relative to expression in thymus and normalized to the expression of GAPDH. Each bar represents the average ± SD of three replicates. (**B**) Real-time PCR analysis of Ik11 transcripts in magnetic bead-purified human leukocyte subsets (CD4^+^ and CD8^+^ T cells, CD19^+^ B cells and CD14^+^ monocytes). The Ik11 levels are expressed as fold change relative to expression in PBLs obtained from the healthy donor #1 (see Figure S3) and normalized to the expression of GAPDH. Each bar represents the average ± SD of three replicates. (**C**) Sequencing of Ik11 real-time PCR products from lymph nodes. The electropherogram shows the sequence corresponding to the junction fragments half exon 5/half exon 8.

Next, the existence of this novel Ikaros splice variant was confirmed. A significant expression of *Ik11* was found in lymph nodes, and less was found in the spleen and thymus, whereas no expression was observed in bone marrow (Figure 2A). These data suggest that this short isoform of Ikaros could have physiological functions in peripheral lymphocytes.

To further characterize *Ik11* expression in PBL sub-populations, CD4^+^, CD8^+^, CD14^+^ and CD19^+^ cells were isolated. *Ik11* was mainly expressed in lymphocyte fractions (Figure 2B). *Ik11* amplification products have been confirmed by sequencing (Figure 2C).

### Ik-11 Functions as a Dominant Negative Isoform of Ikaros

**Figure 3:**
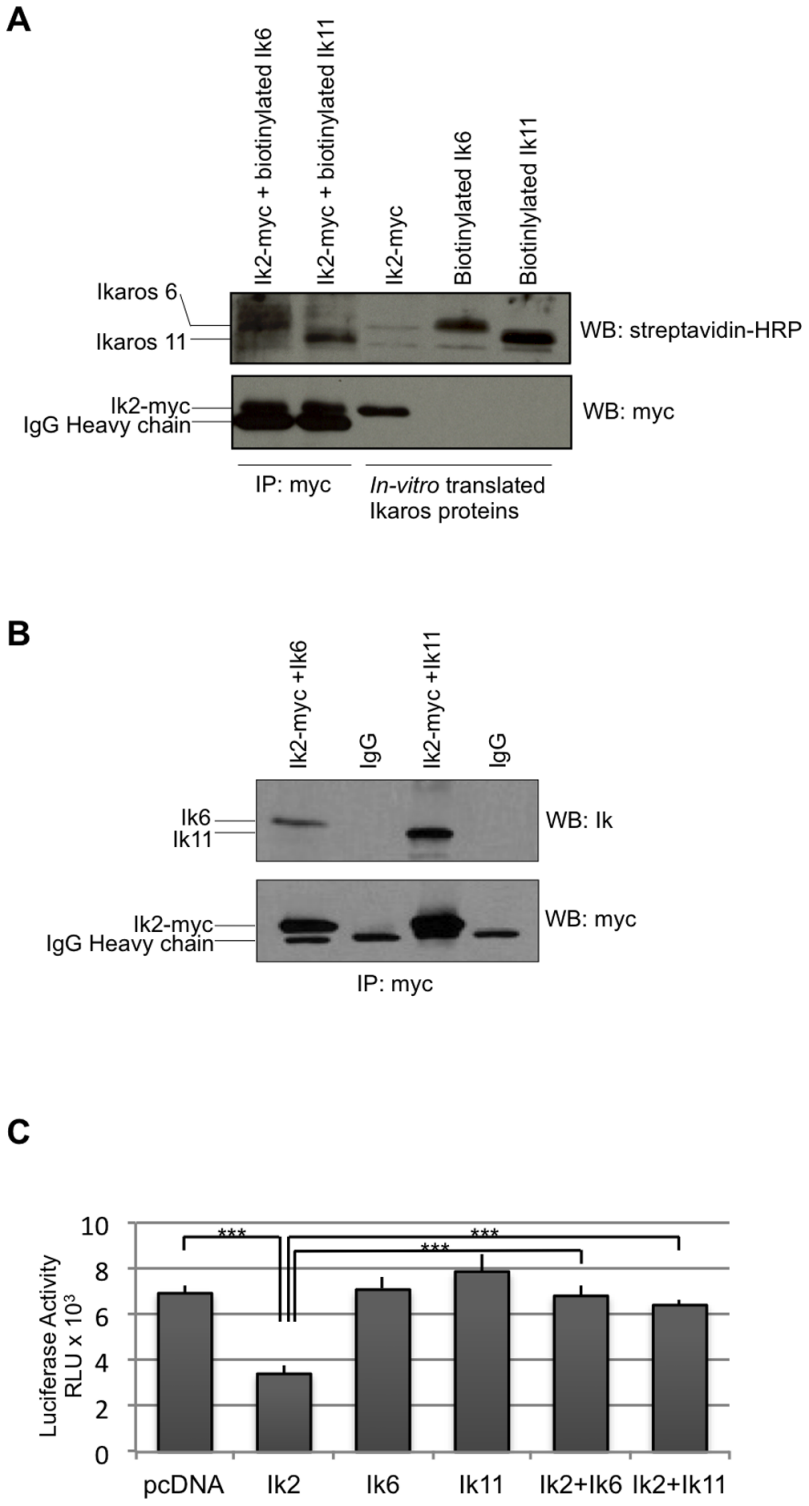
Ik11 acts as a dominant negative isoform. (**A**) *In vitro* co-immunoprecipitation of Ik2 with the short isoforms Ik11 or Ik6. Ik2-myc, biotinylated-Ik11 and biotinylated-Ik6 were generated by *in vitro* transcription/translation. After 1 h incubation of Ik2-Myc with Ik11 or Ik6, the Ik2 complexes were immunoprecipitated with an anti-Myc antibody and subjected to Western blot analysis as indicated (lanes 1 and 2). Lanes 3, 4 and 5 contained the three *in vitro* translated proteins without immunoprecipitation. (**B**) *In vivo* heterodimerization of Ik2 with the splice variants Ik11 or Ik6. The 293T HEK cell line was co-transfected with pcDNA3.1/Myc-HysB-Ik2 along with pcDNA3.1-Ik6 or pcDNA3.1-Ik11. The Ik2 immunoprecipitation was performed with an anti-Myc antibody and the immune-complexes were analyzed by Western blotting as indicated. (**C**) Luciferase assay showing functional dominant-negative activity of Ik11. The 293T HEK cell line was co-transfected with pcDNA3.1-Ik2, pcDNA3.1-Ik6 or pcDNA3.1-Ik11 or their combinations along with a reporter-LUC construct driven by a promoter containing Ikaros-binding sites. Mean ± SD of triplicate wells is shown (***p<0.001). Data shown are representative of three different experiments.

Based on its sequence, Ik11 resembled the DN isoforms of Ikaros proteins (Figure 1A–B). There is evidence in the literature that Ik6, the most studied Ikaros dominant negative isoform, exerts its inhibitory effect by binding the functional splice variants and inhibiting their ability to bind DNA [3], [8], [36]. Therefore, to shed light on Ik11 function, we first analyzed its capability to form heterodimers with the transcriptionally active form Ik2, *in vitro*. Ik2-myc, biotinylated-Ik11 and biotinylated-Ik6 proteins were generated by *in vitro* transcription/translation. Then, co-immunoprecipitations of Ik2-myc with either biotinylated-Ik11 or biotinylated-Ik6 were performed by using an anti-Myc antibody. Immune-complexes were separated by SDS-PAGE and analyzed by streptavidin-HRP Western blot (Figure 3A, first and second lanes). Ik11 was found in Ik2-immunoprecipitated complexes, demonstrating that Ik11 is effectively able to bind Ik2 (Figure 3A, second lane). Ik-2/Ik-6 complexes were shown as a control (Figure 3A, first lane) [1]–[3]. Translated Ikaros proteins were loaded to control the efficiency of the *in vitro* transcription/translation system (Figure 3A, third, fourth and fifth lanes). Anti-Myc Western blot was performed as control of the immunoprecipitation (Figure 3A, lower panel).

We confirmed Ik2/Ik11 interaction by immunoprecipitating protein extracts from 293T cells co-transfected with pcDNA3.1-myc-Ik2 and pcDNA3.1-Ik11, by using an anti-Myc antibody (Figure 3B). Ik11 resulted to be co-immunoprecipitated by the anti-Myc antibody but not by a control IgG. Ik-2/Ik-6 complexes were shown as a control [1]–[3]. This analysis indicates that the interaction between Ik2 and Ik11 also takes place *in vivo*.

Next, the effect of Ik11 on the transcriptional activation of an Ik2-regulated promoter has been evaluated. As expected by the lack of the AD, overexpression of Ik11 alone has no effect on a promoter containing Ikaros consensus sites (Figure 3C). Importantly, Ik11 is able to revert the Ik2-dependent inhibition of the promoter (Figure 3C), demonstrating that Ik11 works as a DN isoform. The choice of a negatively Ik-regulated promoter was due to the fact that most of the known Ik-responsive elements are repressed by this transcription factor, whereas only a few of them are activated.

**Figure 4:**
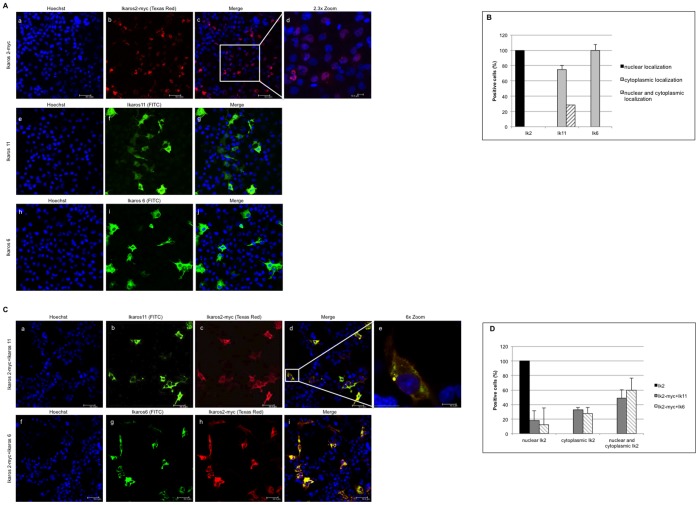
Ik2 subcellular localization changes in presence of Ik11. (**A**) Subcellular localization of Ik2 (panels a–d), Ik11 (panels e-g) and Ik6 (panels h–j). The Cos7 cell line was transfected with pcDNA3.1/Myc-HysB-Ik2, pcDNA3.1-Ik6 or pcDNA3.1-Ik11 constructs and the cellular localization of each isoform was analyzed by confocal microscopy. Immunofluorescence localization of Ik2 was assessed by anti-Myc antibody and Texas Red dye conjugated AffiniPure Goat anti-mouse IgG (H+L) (red fluorescence); Ik6 and Ik11 were detected with anti-Ikaros antibody and Fluorescein (FITC)-conjugated AffiniPure Goat anti-Rabbit IgG (H+L) (green fluorescence). Nuclei were stained with Hoechst 33258 (panels a, e, h, blue fluorescence). Merged images of double fluorescence (Hoechst localization of nuclei plus Ikaros staining) are shown for all of the three isoforms (Ik2: panels c and d, scale bar equals to 50 and 10 microns respectively; Ik11: panel g; Ik6: panel j). x40 magnification (panels a–c, e–j). Panel d was an x2.3 zoom of the white box field indicated in panel c. (**B**) Graphic representation of Ik2, Ik11 and Ik6 subcellular localization. The analysis was conducted counting nuclear or cytoplasmic staining, or both (nuclear+cytoplasmic), of the three isoforms as percent point. 5 fields were counted for each transfection. (**C**) Confocal triple immunofluorescence images of Hoechst 33258 plus Ik2-myc and Ik11 or Ik6. Cos7 cells were co-transfected with either pcDNA3.1/Myc-HysB-Ik2 and pcDNA3.1-Ik11 (a–e) or pcDNA3.1/Myc-HysB-Ik2 and pcDNA3.1-Ik6 (f–i) expression vectors. Staining for Ik11 (green fluorescence), Ik6 (green fluorescence), Ik2 (red fluorescence) and Hoechst 33258 (blue fluorescence) were performed as described in (A). Merged images of triple fluorescence (Hoechst localization of nuclei plus co-localization of Ik2/Ik11 or Ik2/Ik6) were illustrated in panels d-e and panel j, respectively. Scale bar were equals to 50 microns (panels a-d and panels f–j, x40 objective) and 10 microns (panel e, x6 zoom of the white box field indicated in panel d). (**D**) Graphic representation of Ik2 subcellular localization when it is transfected alone or in combination with Ik11 and Ik6. The analysis was conducted counting Ik2 nuclear or cytoplasmic staining, or both (nuclear+cytoplasmic), as percent point. 5 fields were counted for each transfection.

To investigate the mechanism by which Ik11 is able to block Ik2’s transcriptional activity, we looked at the sub-cellular localization of these proteins in the Ikaros-null COS-7 cell line [4], either when overexpressed alone or in combination. The transfection efficiency was evaluated by FACS (Figure S2A). As previously reported in the literature, our immunofluorescence images confirmed that the Ik-2 active isoform localized wholly in the nucleus [1]–[3] (Figure 4A, panels a-d and Figure 4B). Interestingly, the novel DN splice variant Ik-11 showed a predominant cytoplasmic localization (Figure 4A, panels e-g and Figure 4B). A ∼20% of Ik11-transfected cells exhibited both cytoplasmic and nuclear staining, but none of them showed solely nuclear localization of the protein (Figure 4A–B). As known from the literature, Ik-6 localized predominantly in the cytoplasm (Figure 4A, panels h-j and Figure 4B) [4]. Notably, Ik2 changed its localization from the nucleus to the cytoplasm in the presence of Ik11 (Figure 4C, panels a-e and Figure S2B), showing almost a complete merge with this isoform (Figure 4C, panels d, e). Indeed, this translocation was not always complete. In fact, ∼50% of positive cells showed both cytoplasmic and nuclear staining for Ik2-myc in presence of Ik11 (Figure 4C–D and Figure S2B). Cytoplasmic Ik2 was not detectable in cells negative for Ik11. Ik2+Ik11 immunofluorescence data were comparable with those obtained from the co-transfection of Ik6 with Ik2, suggesting that Ik11 resemble Ik6 in its ability of sequestering and inactivating Ik2.

Taken all together, these data demonstrated that Ik11 is a novel Ikaros splice variant, which acts as a dominant negative isoform. Indeed, the ability of Ik11 to block Ik2’s transcriptional functions could be due, at least in part, to Ik11-mediated cytoplasmic sequestration of Ik2.

### Ik-11 Enhances Cell Proliferation and Impairs Apoptosis

Several lines of evidence have suggested that *Ikaros* acts as a tumor suppressor gene [8]. In fact, *Ikaros*-deficient mice revealed that the absence of Ikaros expression, or the presence of DN Ikaros mutants, leads to the development of T-cell leukemia in mice [8]. Moreover, Ikaros provides thresholds that regulate proliferation at key stages of T-cell development. Furthermore, T cells from Ikaros-deficient mice showed facilitated cell-cycle entry in response to minimal TCR engagement and accelerated G_1_-S transition in response to IL2R signaling [37]. In addition, Ikaros suppresses pre-B cell proliferation, thus allowing for a correct differentiation program [11]. Loss of Ikaros activity is indeed observed in more than 80% of Ph^+^ ALL [38]. Ikaros proteins also play an important role in monocyte/macrophage development [4].

**Figure 5:**
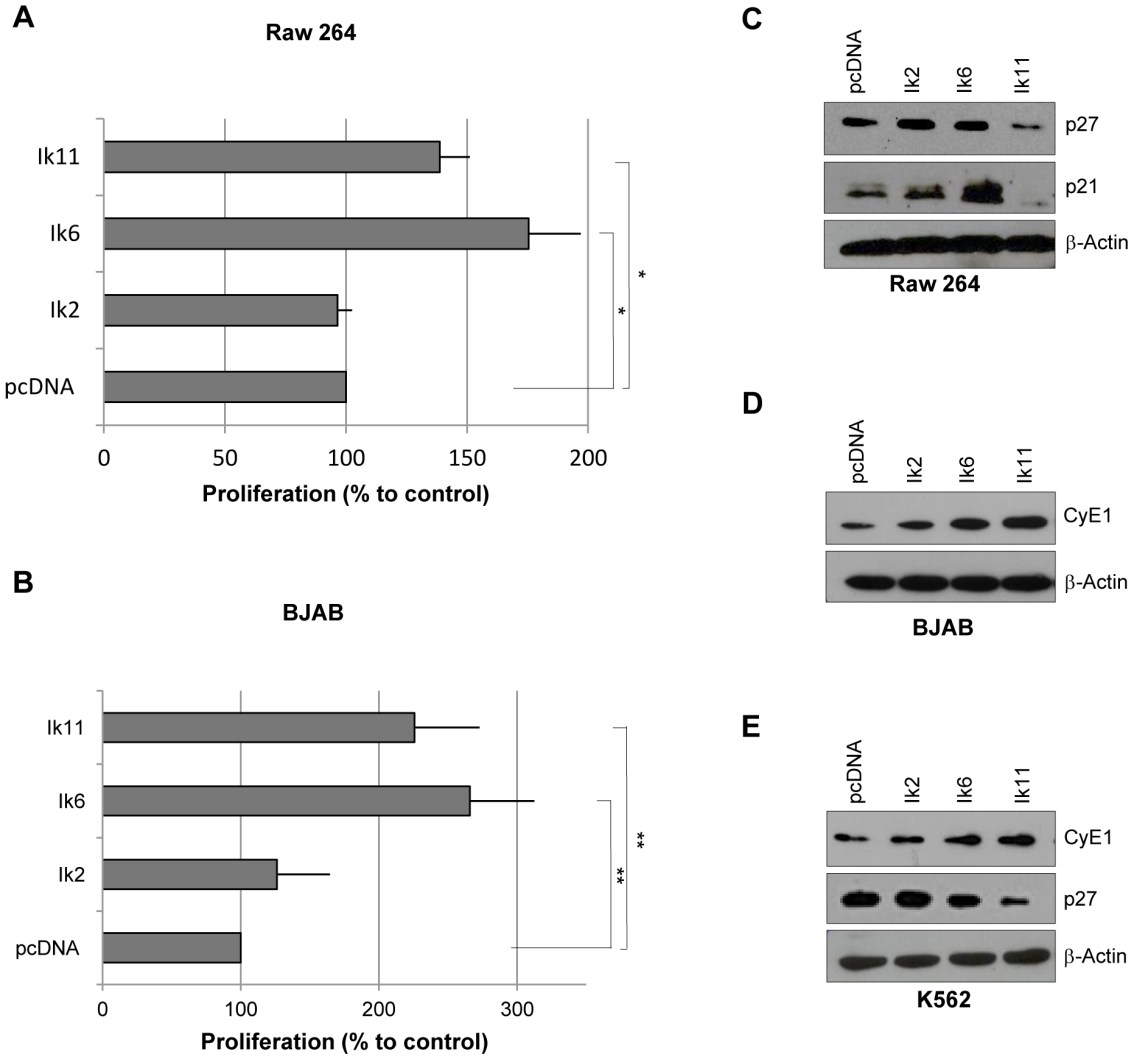
Ik11 overexpression promotes cell proliferation. (**A–B**) Analysis of cell proliferation in Raw 264 (A) and BJAB (B) cell lines. Cells were transfected with pcDNA3.1-Ik2, pcDNA3.1-Ik6, pcDNA3.1-Ik11 or empty vector. Proliferation was determined by the MTS assay at 24 h (BJAB) or 48 h (RAW 264) and calculated as the percent of control (pcDNA3.1). Mean ± SD is shown (*p<0.05; **p<0.001). Data shown are representative of three different experiments. (**C–E**) Western blot analysis of p27, p21 and cyclin E in RAW 264 (C), BJAB (D) and K562 (E) cell lines.

The overexpression of DN Ikaros isoforms impaired the growth-inhibitory function of Ikaros in several systems [39]–[41]. Therefore, we tested whether the novel DN Ik11 isoform was capable of promoting cell proliferation. Transient overexpression of Ik2 did not impact Raw 264 cell growth, whereas transfection of Ik11 induced a significant increase of cell proliferation (Figure 5A). The same results have been obtained by using the BJAB cell line (Figure 5B). Increased proliferation by Ik11 overexpression was associated with a down-regulation of cyclin-dependent kinase inhibitors p21 and p27 in Raw264 cells (Figure 5C), as well as with an up-regulation of cyclin E in BJAB cells (Figure 5D). A decrease of p27 protein levels, as well as the up-regulation of cyclin E was also detected in K562 cells overexpressing Ik11 (Figure 5E). The proliferation rate induced by Ik11 overexpression was similar to that obtained upon Ik6 transfection in both cell lines (Figure 5A–B). High cyclin E levels were also observed in the Ik6-transfected BJAB cell line, while no down-regulation of p21 and p27 was found in Ik6-expressing Raw264 and K562 cell lines, suggesting that Ik11 and Ik6 might promote proliferation through different mechanisms.

Ikaros is also known to play a role in the control of apoptosis. Bone marrow erythroid cells from Ikaros-null mice were less susceptible to oxidative stress-induced apoptosis than control cells [12]. Moreover, apoptosis was increased upon overexpression of full-length Ikaros in leukemic cell lines [13]. Furthermore, the overexpression of Ik6 can delay apoptotic cell death upon growth factor withdrawal in myeloid and lymphoid cytokine-dependent cell lines and confer resistance to dexamethasone and anti-IgM-induced apoptosis in B cells [42], [43].

**Figure 6:**
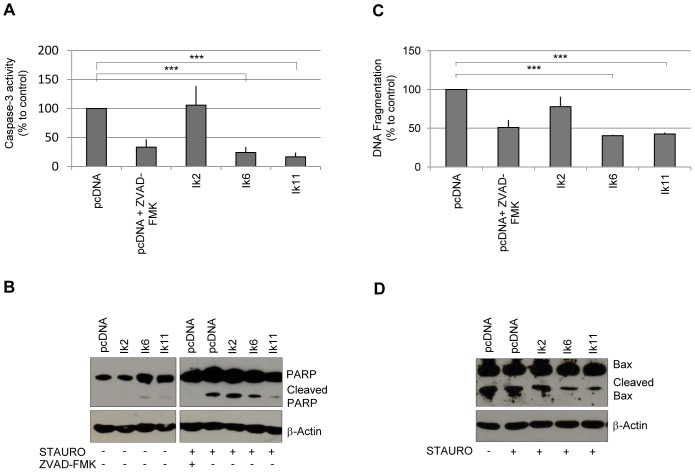
Ik11 overexpression protects against apoptosis. Evaluation of apoptosis by measurement of caspase-3 activity (**A**), examination of PARP cleavage products (**B**) and determination of mono- and oligo-nucleosome enrichment (**C**). Raw 264 cells were transfected with pcDNA3.1-Ik2, pcDNA3.1-Ik6, pcDNA3.1-Ik11 or empty vector and were incubated with 100 nM staurosporine or vehicle for 14 hours. Empty vector-transfected cells were also treated with 50 µM Z-VAD-FMK, a general caspase inhibitor. Values were calculated as percent of control (pcDNA3.1). Mean ± SD is shown (***p<0.0001) (A,C). Data shown are representative of three different experiments. (**D**) Western blot analysis of Bax protein. Raw 264 cells were transfected with Ikaros isoforms and treated with staurosporine as previously described. Protein levels of Bax and Bax/p18 cleavage products are shown.

Therefore, we assessed the role of Ik11 in staurosporine-induced apoptosis. As shown in Figure 6, overexpression of Ik11 strongly protected Raw264 cells against staurosporine-induced apoptosis (Figure 6A–C). In this cell context, Ik11 protected against staurosporine-induced apoptosis by inhibiting Bax cleavage, with the consequent decrease of the potent proapoptotic molecule p18-Bax (Figure 6D).

Therefore, Ik11 expression impacts both cell proliferation and cell death, suggesting that this new DN Ikaros isoform might play a role in the development of hematological cancers.

### Ik11 is Aberrantly Expressed in B-cell Lymphoproliferative Disorders

**Figure 7:**
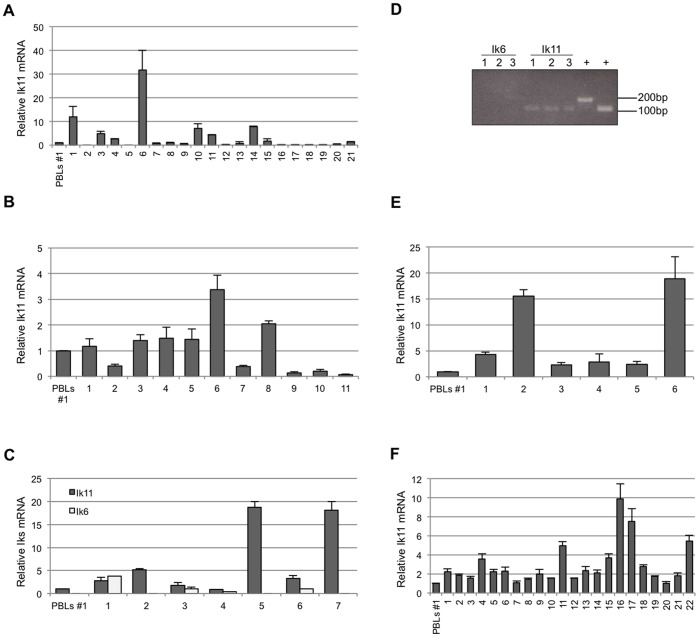
Increased *Ik11* expression in several hematological malignancies. (**A, B, C**) Real-time PCR analysis of *Ik11* and *Ik6* mRNAs in samples of chronic myeloid leukemia (A, n = 21), acute lymphoblastic leukemia (B, n = 11) and myelodysplastic syndromes (C, n = 7). The Ik11 levels are expressed as fold change relative to expression in PBLs obtained from the healthy donor #1 (see Figure S3) and normalized to the expression of GAPDH. Each bar represents the average ± SD of three replicates. (**D**) Expression of *Ik11* and *Ik6* in commercial samples of lymphoproliferative disorders (n = 3). 1 = Poorly differentiated malignant lymphoma; 2 = Hodgkin’s lymphoma; 3 = non-Hodgkin’s lymphoma, diffuse; + = PCR positive controls. (**E, F**) Real-time PCR analysis of *Ik11* and *Ik6* mRNAs in samples of lymphoma (E, n = 7) and chronic lymphoblastic leukemia (F, n = 22). The Ik11 levels are expressed as fold change relative to expression in PBLs obtained from the healthy donor #1 (see Figure S3) and normalized to the expression of GAPDH. Each bar represents the average ± SD of three replicates.

Aberrant expression of DN Ikaros isoforms, particularly Ik6, has been found in adult B cell ALL [22], [27], as well as in myelodysplastic syndrome [44], AML [4] and adult and juvenile CML [29]. Therefore, we investigated the involvement of the novel DN Ik11 in hematological tumors. Expression of Ik11 in PBLs was used as reference value. To this end, we evaluated Ik11 mRNA levels in 10 different PBLs samples obtained from healthy donors, proving a similar expression values in all samples (Figure S3). Therefore, we assessed the expression of *Ik11* in several lymphoid and myeloid cell lines, detecting no significant increase with respect to PBLs (Figure S4). Next, we analyzed *Ik11* mRNA in those hematological disorders in which Ik6 overexpression was previously demonstrated [4], [22], [26], [27], [29], [44]. In particular, our analysis focused on 21 samples of CML (Figure 7A and Table S1), 11 samples of ALL (Figure 7B and Table S2) and 7 samples of myelodysplastic syndromes (Figure 7C and Table S3). *Ik11* mRNA was increased more than 2-fold in 7 of 21 (31.8%) samples of CML (Figure 7A) and in 2 of 11 (18.2%) samples of ALL (Figure 7B). Notably, Ik6 expression was not detectable in all CML and ALL samples analyzed (see discussion). *Ik11* expression was also increased in 5 of 7 (71.4%) samples of myelodysplastic syndromes (Figure 7C). Nevertheless, the relevance of this latter data needs to be confirmed by using a wider set of myelodysplastic samples.

Finally, we analyzed *Ik11* expression in the B-cell lymphoproliferative disorders in which DN Ikaros isoforms have not been reported to play a role. The mRNA of three commercially available samples of lymphoma (Figure 7D), six samples of Hodgkin’s and non-Hodgkin’s lymphoma (Figure 7E and Table 1) and 22 samples of CLL (Figure 7F and Table 2) have been analyzed. Conventional semi-quantitative PCR demonstrated that *Ik11* was expressed in the three commercially available samples of lymphoma (Figure 7D). *Ik11* PCR-amplified products were then confirmed by sequencing (Figure S5). Indeed, increased expression of *Ik11* was found in all lymphoma samples (n = 6) (Figure 7E), with two samples showing strikingly high levels of *Ik11* (Figure 7E). Moreover, *Ik11* was also increased in 12 of 22 CLL samples (54.5%) (Figure 7F). Given that the unmutated *IgVH* gene status or some cytogenetic abnormalities are associated with a worse clinical outcome, we analyzed the level of *Ik11* expression in these CLL subgroups. No correlations of these clinical markers with Ik11 were observed. Notably, the highest level of *Ik11* expression was observed in those patients (cases 16, 17 and 22) with rapidly progressive disease (cases 16 and 22 for leukocytosis and case 17 for anemia and thrombocytopenia). Remarkably, no signal for Ik6 was revealed in both lymphoma and CLL samples.

**Table 1:** Clinical data in patients with Lymphoma.

N°	Sex	Age	WHO	Stage	IPI/FLIPI or IPS	Karyotype	Disease status at sampling	Outcome
1	F	69	Diffuse Large B-cell Non-Hodgkin lymphoma	III A	2	NA	Relapse	Alive
2	F	85	Diffuse Large B-cell Non-Hodgkin lymphoma	II E	1	NA	Relapse	Dead
3	M	36	Hodgkin lymphoma	III B	2	NA	Diagnosis	Alive
4	F	85	Diffuse Large B-cell Non-Hodgkin lymphoma	II A	3	NA	Diagnosis	Dead
5	M	63	Diffuse Large B-cell Non-Hodgkin lymphoma	III B	5	Normal	Diagnosis	Dead
6	F	89	Marginal Zone Non-Hodgkin lymphoma	II	1	NA	Diagnosis	Dead

NA indicates not available.

**Table 2:** Clinical data in patients with Chronic Lymphocytic Leukemia.

N°	Sex	Age	Disease stage at diagnosis	V_H_ gene status	Treatment	Karyotype	WBC/mmc	% CD19^+^/CD5^+^
1	F	63	II	Unmut	No	Del 13	82600	85%
2	M	78	IV	Mut	Yes	Del 13q14; Del 17p	117000	76%
3	M	66	II	Mut	Yes	NA	32000	65%
4	F	60	I	Mut	No	normal	80000	NA
5	F	75	III	Mut	No	trisomy 12	74080	80%
6	M	73	NA	Mut	Yes	normal	122400	77%
7	M	78	I	Unmut	Yes	Del 11q	64000	15%
8	F	85	I	Mut	Yes	NA	46180	76.70%
9	M	56	II	Mut	No	normal	13450	40%
10	M	82	II	Unmut	Yes	normal	21630	74.20%
11	F	76	0	Mut	No	trisomy 12	12000	20%
12	F	71	0	Mut	No	NA	14670	23%
13	F	81	0	Mut	No	NA	19570	60%
14	F	52	0	Mut	No	Del 13q14	44100	84%
15	F	59	I	Mut	No	normal	19580	62%
16	M	55	I	Mut	Yes	normal	81220	88%
17	M	81	I	Mut	Yes	Del 13	18460	80%
18	F	80	0	Mut	no	normal	17860	49%
19	M	66	0	Mut	no	NA	23160	59%
20	M	66	II	Mut	no	NA	11890	58%
21	M	60	II	Mut	Yes	normal	16250	NA
22	M	74	0	NA	Yes	Del 13	104000	96%

NA indicates not available.

Taken together, all these data indicated that *Ik11* aberrant expression is strongly associated with B-cell lymphoproliferative disorders and, to a lesser degree, with B-ALL and CML.

## Discussion

During the past years, Ikaros has been established as one of the most clinically relevant tumor suppressors in several hematological malignancies. Expression of DN isoforms is associated with adult B-cell ALL, as well as with myelodysplastic syndrome, AML, and adult and juvenile CML. In addition, multiple microarray-based analyses of genetic changes and alterations in gene expression have revealed that Ikaros plays a key role in tumor suppression in pediatric B-cell ALL. Indeed, a modest decrease in Ikaros activity is sufficient to contribute to leukemogenesis [3], [4], [17], [19]–[27], [29], [44].

In this study we reported the isolation of a novel, non-canonical, Ikaros DN isoform, named Ik11. Ik11 protein has two C-terminal and only one N-terminal zinc-finger domains, thus lacking the functional DNA binding domain, but able to form homo- and heterodimers. All known DN Ikaros isoforms share these structural characteristics. However, in contrast to all other DN Ikaros proteins, Ik11 completely skips the AD due to alternative splicing. To our knowledge, this is the first evidence of a non-canonical splice variant of Ikaros. How the absence of the AD can impact on Ik11 protein structure–ie., tertiary folding, stability, phosphorylation acceptor sites etc.–needs to be established with further experiments. Nevertheless, we demonstrated that Ik11 functionally acts as a DN protein. In fact, Ik11 is able to block the activity of transcriptionally active isoforms at least in part by binding them and inducing their cytoplasmic sequestration, as previously demonstrated also for other DN Ikaros isoforms [4].

Notably, Ik11 was aberrantly expressed in B-cell lymphoproliferative disorders, such as CLL. This disease is characterized by the monoclonal expansion of B lymphocytes in the peripheral blood, bone marrow and lymphoid organs with an indolent course that can become aggressive or even fatal [45]. The pathogenic events of CLL are not well known. Here, we showed that Ik11 is overexpressed in 12 of 22 (54.5%) cases of CLL, with the highest expressions of Ik11 observed in those patients in a rapidly progressed disease state. To our knowledge, this is the first evidence of aberrant expression of Ikaros DN isoforms in B-cell lymphoproliferative disorders. An increased expression of Aiolos, another member of Ikaros family, has been recently demonstrated in CLL. The authors showed that Aiolos overexpression confers a survival advantage to the CLL population [46]. In CLL cells, whether Ik11 and Aiolos play distinct pathogenic roles or they work in concert forming a heterodimer remains to be determined.

The most studied dominant-negative isoform of Ikaros is Ik6, whose aberrant expression has been found in adult B-cell ALL [22], [27], as well as in myelodysplastic syndrome [44], AML [4] and adult and juvenile CML [29]. Therefore, we also analyzed the expression of a DN Ik6 isoform in our hematological cancer samples. Surprisingly, we did not detect Ik6 mRNA in any case, except for four samples of myelodysplastic syndromes. Conflicting data have been reported in the literature on the frequency of Ik6 expression in hematological cancers. Our data are in accordance with previous data on U.S. patients reported by Sun and colleagues [23]–[25], but in contrast with other studies on Japanese and Chinese patients [20]–[22], [26], [27], [29]. The differences between the Japanese and Chinese reports on the one hand and our and Sun’s studies on the other hand could be explained by ethnic diversity, as already suggested by Takanashi et al. [21].

Given the large amount of research on Ikaros DN isoforms in cancer, it might seem surprising that Ik11 was not previously identified. However, *Ikaros* expression has most frequently been analyzed by nested-PCR, using a single primer pair common to all isoforms. In many of these works, the antisense primer annealed to the first part of exon 8, corresponding to the AD, which is missing in the *Ik11* transcript [19]–[29], [47]. In a previous work by Iacobucci and colleagues [16], the detection of *Ikaros* isoforms was performed by PCR followed by capillary electrophoresis, a highly sensitive, accurate and standardized method. Also in this case, the reverse primer was complementary to the exon 8 region, which is absent in *Ik11*. However, *Ik11* has not been detected by PCR even when a primer pair complementary to the start and the stop codons of full-length *Ikaros* was used [22]. A possible explanation is that a single PCR reaction may not have sufficient sensitivity to detect all different isoforms, because some transcripts may amplify more efficiently than others. Therefore, the existence of this novel non-canonical splice variant of Ikaros indicates that screening methods to detect and quantify Ikaros short splice variants missing the AD needs to be performed.

The expression of *Ik11* is not restricted to malignant cells, as also demonstrated for other DN isoforms [16], suggesting that alternative splicing of the *Ikaros* gene might be responsible for the generation of a complex regulatory network that controls normal hematopoiesis. Our expression pattern analysis showed that *Ik11* mRNA is mainly present in lymph nodes and, to a lesser extent, in spleen and thymus. Yet, *Ik11* is mainly expressed in B- and T-cell fractions of PBLs, indicating that it could have physiological roles in peripheral lymphocytes. Further experiments are necessary to better define this issue.

Ikaros DN isoforms have been shown to affect cell proliferation. Ectopic expression of Ik6 can immortalize murine hematopoietic progenitor cells in myeloid conditions resulting in growth factor-independent proliferation of a myeloid cell line [4], [39]. Our results showed that overexpression of Ik11 enhances cell proliferation of both myeloid and lymphoid cell lines by modulating the protein levels of three of the major actors involved in the G1 checkpoint and G1-S transition, such as p21 and p27 cyclin-dependent kinase inhibitors and cyclin E. Interestingly, p27 and cyclin E deregulation has been already found in lymphomas [48].

Apoptosis escape has been indicated as another relatively common mechanism by which Ikaros DN proteins can promote oncogenesis [4]. Overexpression of the Ik6 DN isoform in myeloid and lymphoid cytokine-dependent cell lines can delay apoptosis upon growth factor withdrawal [41] and results in the modulation of Bcl-2 family members in different systems [4], [41]. We found that Ik11 strongly protects Raw264 from staurosporine-induced apoptosis by inhibiting Bax cleavage and the consequent generation of the potent pro-apoptotic molecule p18-Bax.

Unlike Ik11 and Ik6, the overexpression of Ik2 seems to have no significant effects on both cell proliferation and apoptosis. Therefore, the modulation of cell cycle- and apoptosis-related proteins by these short isoforms could not be entirely related to their ability to inhibit the active Ikaros isoforms, leading to the hypothesis that other mechanisms in terms of gene regulation are involved. Recently, it has been demonstrated that Ik6 can prompt survival of pituitary tumor cells by acetylating Bcl-X_L_ promoter and that this effect was not mediated entirely by disruption of Ik1 action [41]. Thus, it seems that not only the full-length isoforms, but also the short ones can influence gene expression by means of epigenetic mechanisms; it remains to be clarified whether also Ik11 can directly induce epigenetic modifications.

In conclusion, our data identify Ik11 as a novel Ikaros DN isoform generated by non-canonical splicing. Aberrant expression of Ik11 was mainly found in CLL and lymphomas. Ik11 overexpression interferes with both proliferation and apoptotic pathways, providing a mechanism for DN Ik11 isoform involvement in human hematological malignancies, primarily in B-cell lymphoproliferative disorders. Taken together, these findings suggest that Ik11 could represent a novel marker for CLL.

## Supporting Information

Figure S1 **Nucleotide sequence alignment of the full-length** ***Ik1*** **and** ***Ik11*** **.** Nucleotide sequence alignment of the full-length Ik1 and the novel isoform Ik11. The start of each exon are represented by grey letters.
(TIF)

Figure S2 **Ik2 subcellular localization changes in presence of Ik11.** (A) Cos7 cells were transfected with peGFP vector and transfection efficiency was evaluated by FACS analysis. (B) Confocal triple immunofluorescence images of Hoechst 33258 plus Ik2-myc and Ik11. Cos7 cells were co-transfected with pcDNA/Myc-HysB-Ik2 and pcDNA3.1-Ik11. Staining for Ik11 (green fluorescence), Ik2 (red fluorescence) and Hoechst 33258 (blue fluorescence) were performed as described in Figure 4A. Scale bars were equals to 50 microns (panels a–d, x40 objective) and 10 microns (panels e–h, x6.5 zoom of the white box field indicated in panels a–d).
(TIF)

Figure S3 **IK11 expression in hPBLs from healthy donors.** Real-time PCR analysis of *Ik11* mRNAs in 10 different samples of hPBLs obtained from healthy donors. The Ik11 levels are expressed as fold change relative to expression in PBLs #1 and normalized to the expression of GAPDH. Each bar represents the average ± SD of three replicates.
(TIF)

Figure S4 **IK11 expression in myeloid and lymphoid cell lines.** Real-time PCR analysis of *Ik11* and *Ik6* mRNAs in myeloid and lymphoid cell lines (see Supporting Text S1). The Ik11 levels are expressed as fold change relative to expression in PBLs obtained from the healthy donor #1 (see Figure S3) and normalized to the expression of GAPDH. Each bar represents the average ± SD of three replicates.
(TIF)

Figure S5 ***Ik11*** **PCR-amplified products were confirmed by sequencing.** Sequencing of *Ik11* semi-quantitative PCR products shown in Figure 6D. The electropherograms show the sequences corresponding to the junction fragments exon 3/exon 5 and half exon 5/half exon 8.
(TIF)

Table S1 **Clinical data in patients with Chronic Myeloid Leukemia.**
(TIF)

Table S2 **Clinical data in patients with Acute Lymphoblastic Leukemia.**
(TIF)

Table S3 **Clinical data in patients with Myelodysplastic Syndromes.**
(TIF)

Text S1 **Supplementary Material and Methods.**
(DOCX)
