# Supplementary figures and images for: A Novel, Non-canonical Splice Variant of the Ikaros Gene Is Aberrantly Expressed in B-cell Lymphoproliferative Disorders
Source: PLoS One. 2013 Jul 9;8(7):e68080. doi: 10.1371/journal.pone.0068080 (PMC3706598; doi:10.1371/journal.pone.0068080)

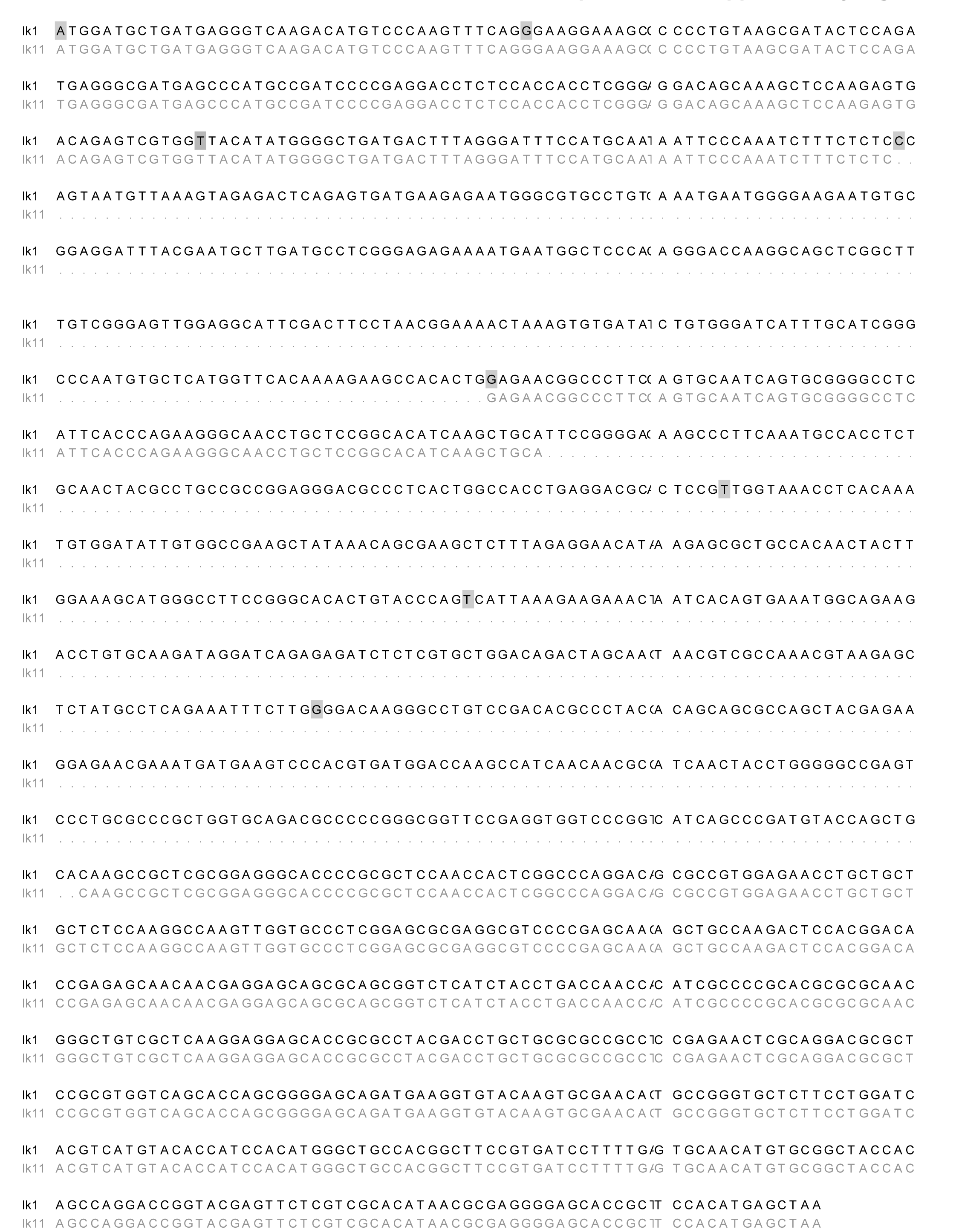

Supplement: Figure S1 — Nucleotide sequence alignment of the full-length Ik1 and Ik11 . Nucleotide sequence alignment of the full-length Ik1 and the novel isoform Ik11. The start of each exon are represented by grey letters. (TIF) [file pone.0068080.s001.tif]

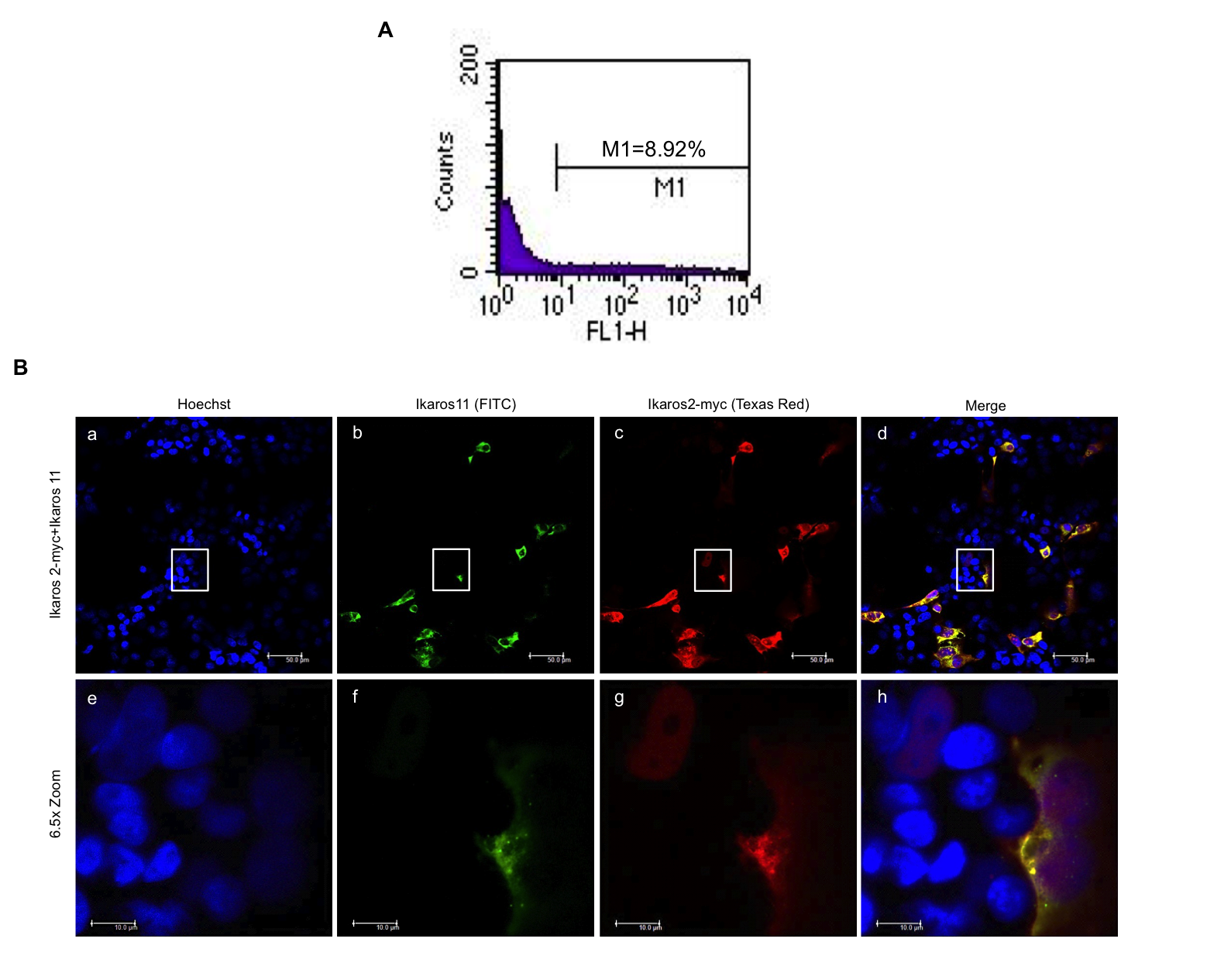

Supplement: Figure S2 — Ik2 subcellular localization changes in presence of Ik11. (A) Cos7 cells were transfected with peGFP vector and transfection efficiency was evaluated by FACS analysis. (B) Confocal triple immunofluorescence images of Hoechst 33258 plus Ik2-myc and Ik11. Cos7 cells were co-transfected with pcDNA/Myc-HysB-Ik2 and pcDNA3.1-Ik11. Staining for Ik11 (green fluorescence), Ik2 (red fluorescence) and Hoechst 33258 (blue fluorescence) were performed as described in Figure 4A. Scale bars were equals to 50 microns (panels a–d, x40 objective) and 10 microns (panels e–h, x6.5 zoom of the white box field indicated in panels a–d). (TIF) [file pone.0068080.s002.tif]

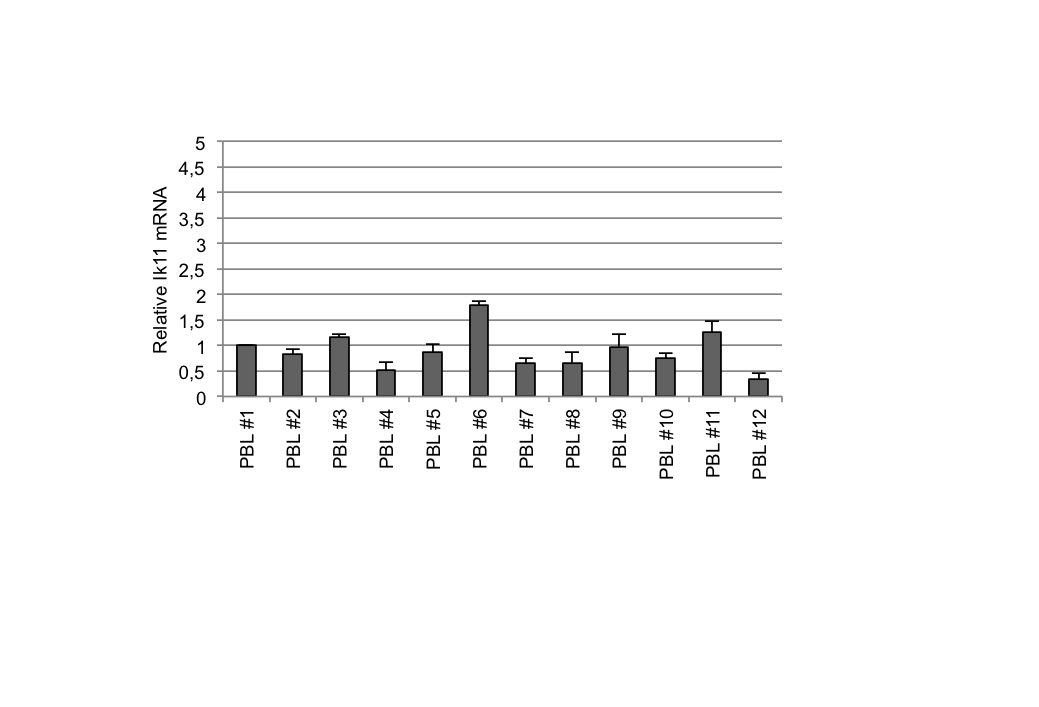

Supplement: Figure S3 — IK11 expression in hPBLs from healthy donors. Real-time PCR analysis of Ik11 mRNAs in 10 different samples of hPBLs obtained from healthy donors. The Ik11 levels are expressed as fold change relative to expression in PBLs #1 and normalized to the expression of GAPDH. Each bar represents the average ± SD of three replicates. (TIF) [file pone.0068080.s003.tif]

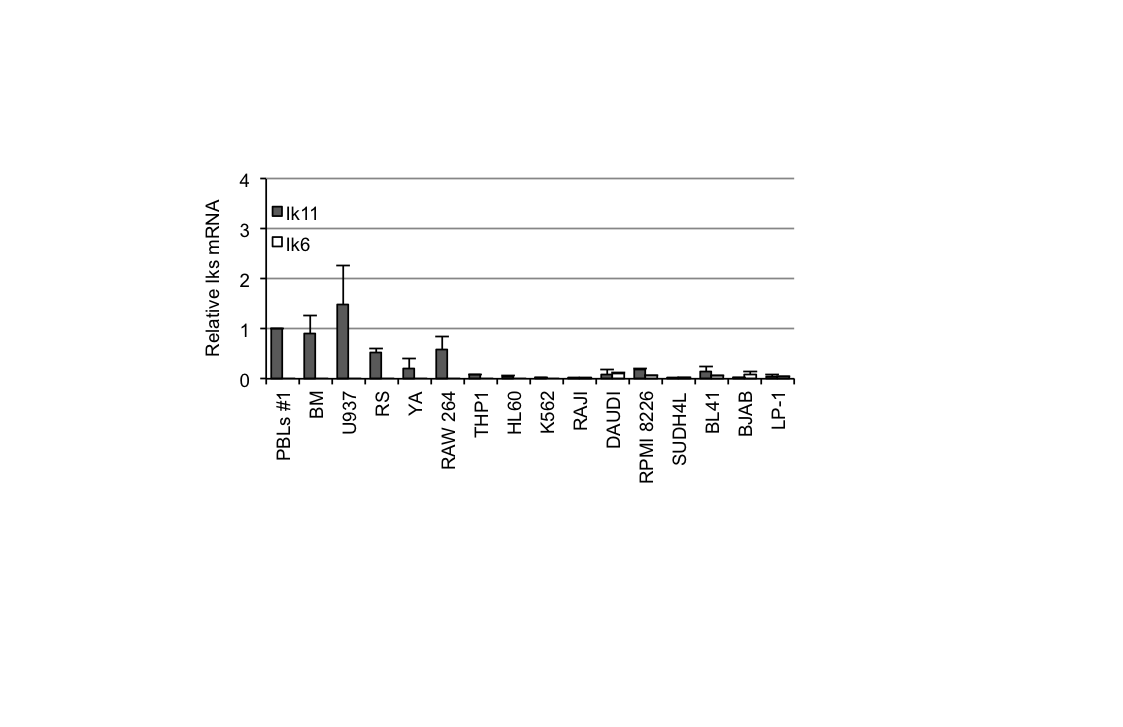

Supplement: Figure S4 — IK11 expression in myeloid and lymphoid cell lines. Real-time PCR analysis of Ik11 and Ik6 mRNAs in myeloid and lymphoid cell lines (see Supporting Text S1). The Ik11 levels are expressed as fold change relative to expression in PBLs obtained from the healthy donor #1 (see Figure S3) and normalized to the expression of GAPDH. Each bar represents the average ± SD of three replicates. (TIF) [file pone.0068080.s004.tif]

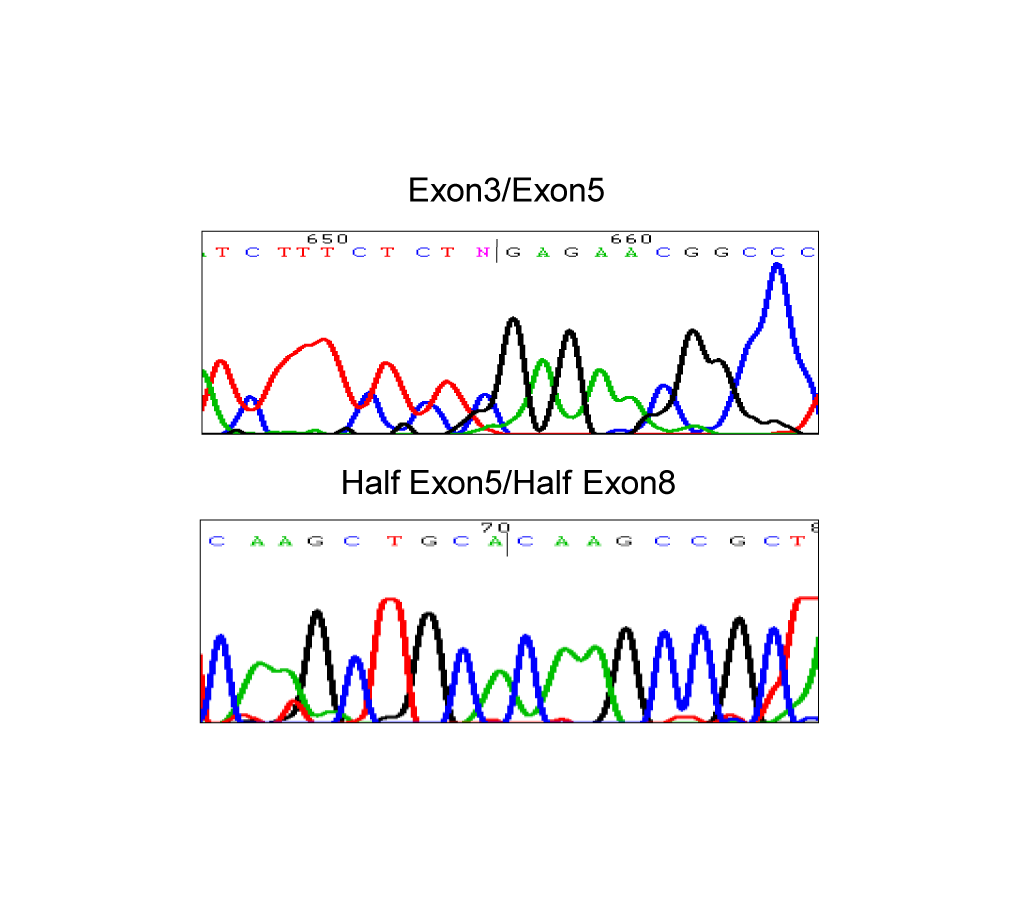

Supplement: Figure S5 — Ik11 PCR-amplified products were confirmed by sequencing. Sequencing of Ik11 semi-quantitative PCR products shown in Figure 6D. The electropherograms show the sequences corresponding to the junction fragments exon 3/exon 5 and half exon 5/half exon 8. (TIF) [file pone.0068080.s005.tif]

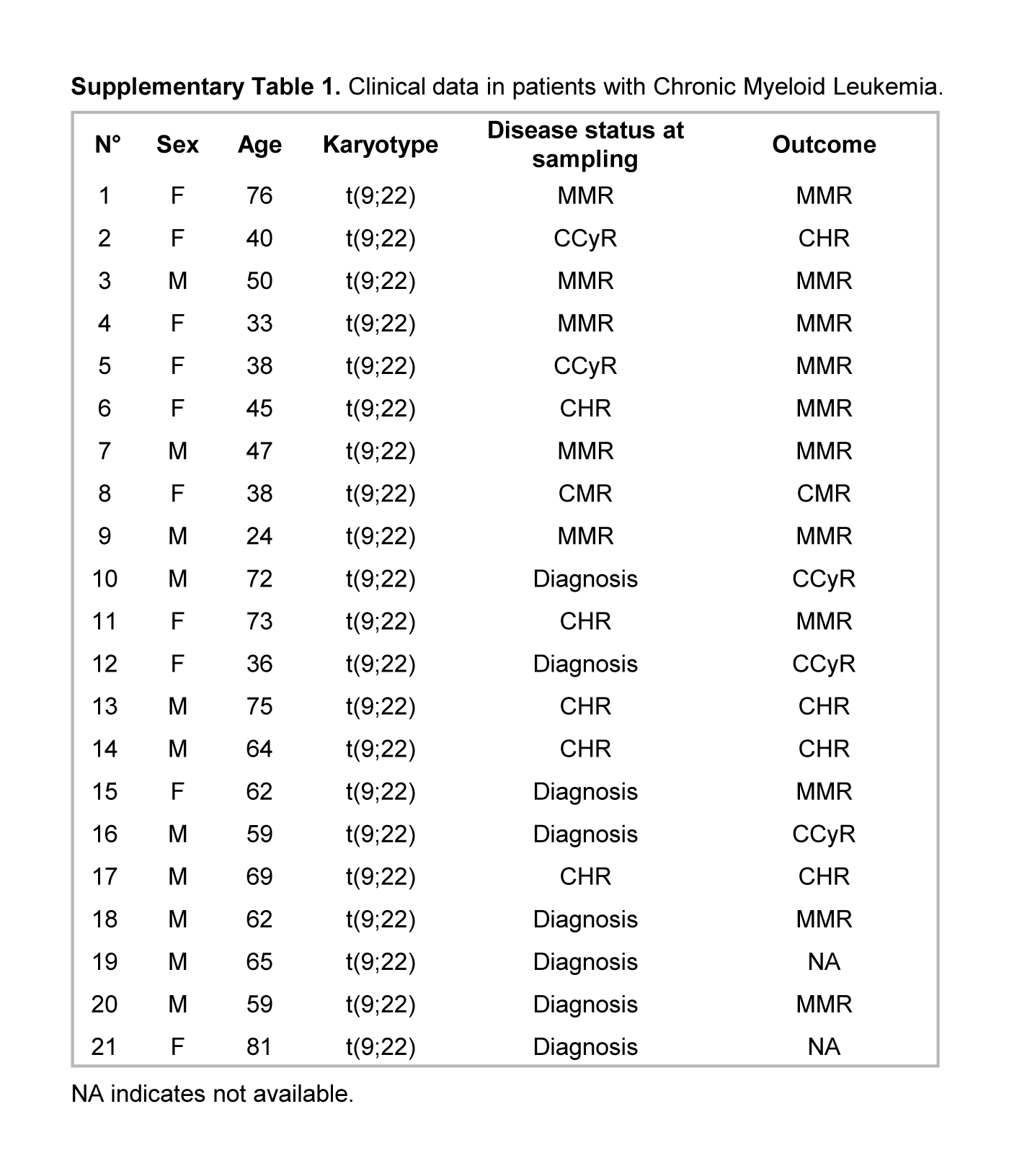

Supplement: Table S1 — Clinical data in patients with Chronic Myeloid Leukemia. (TIF) [file pone.0068080.s006.tif]

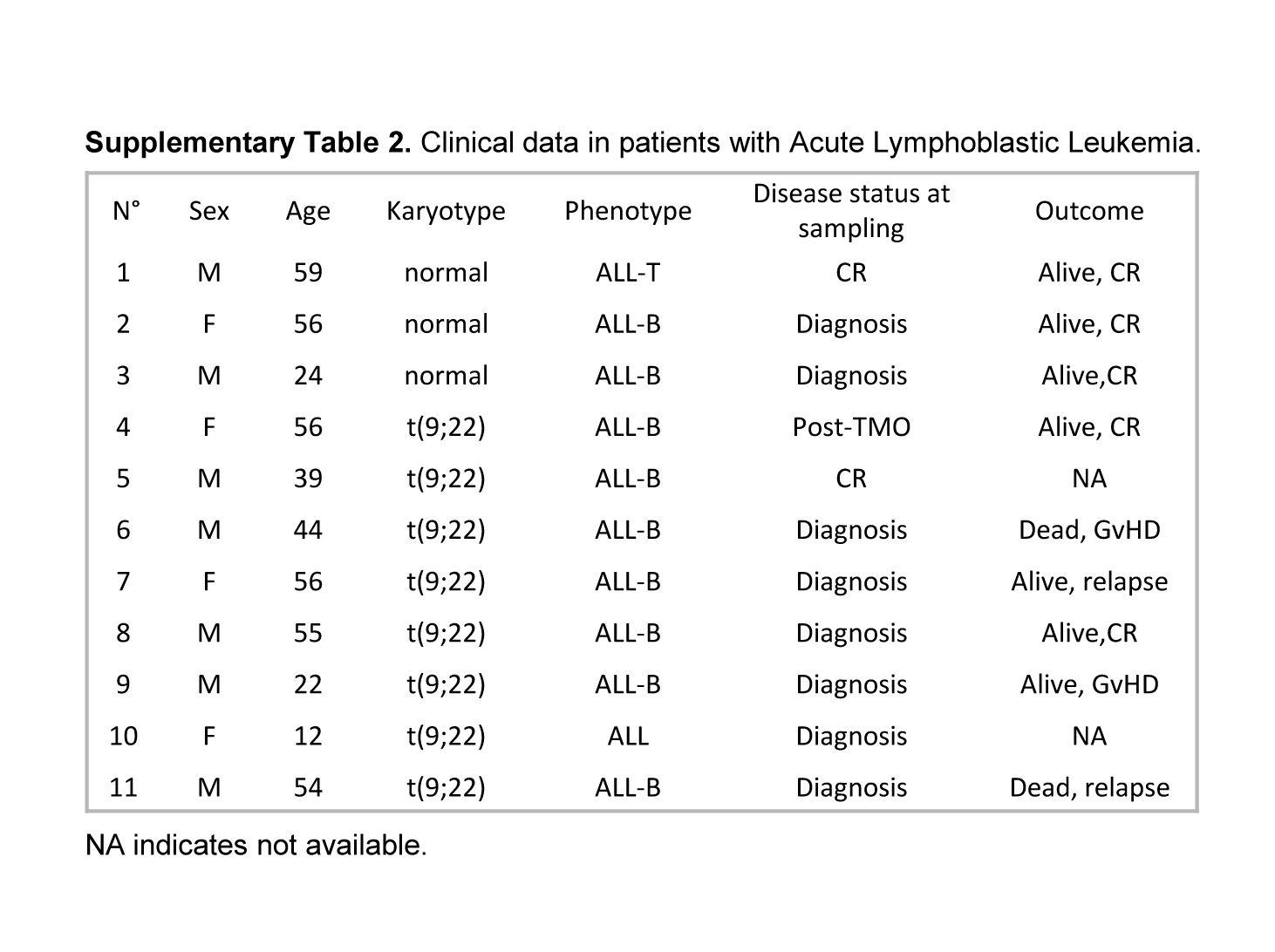

Supplement: Table S2 — Clinical data in patients with Acute Lymphoblastic Leukemia. (TIF) [file pone.0068080.s007.tif]

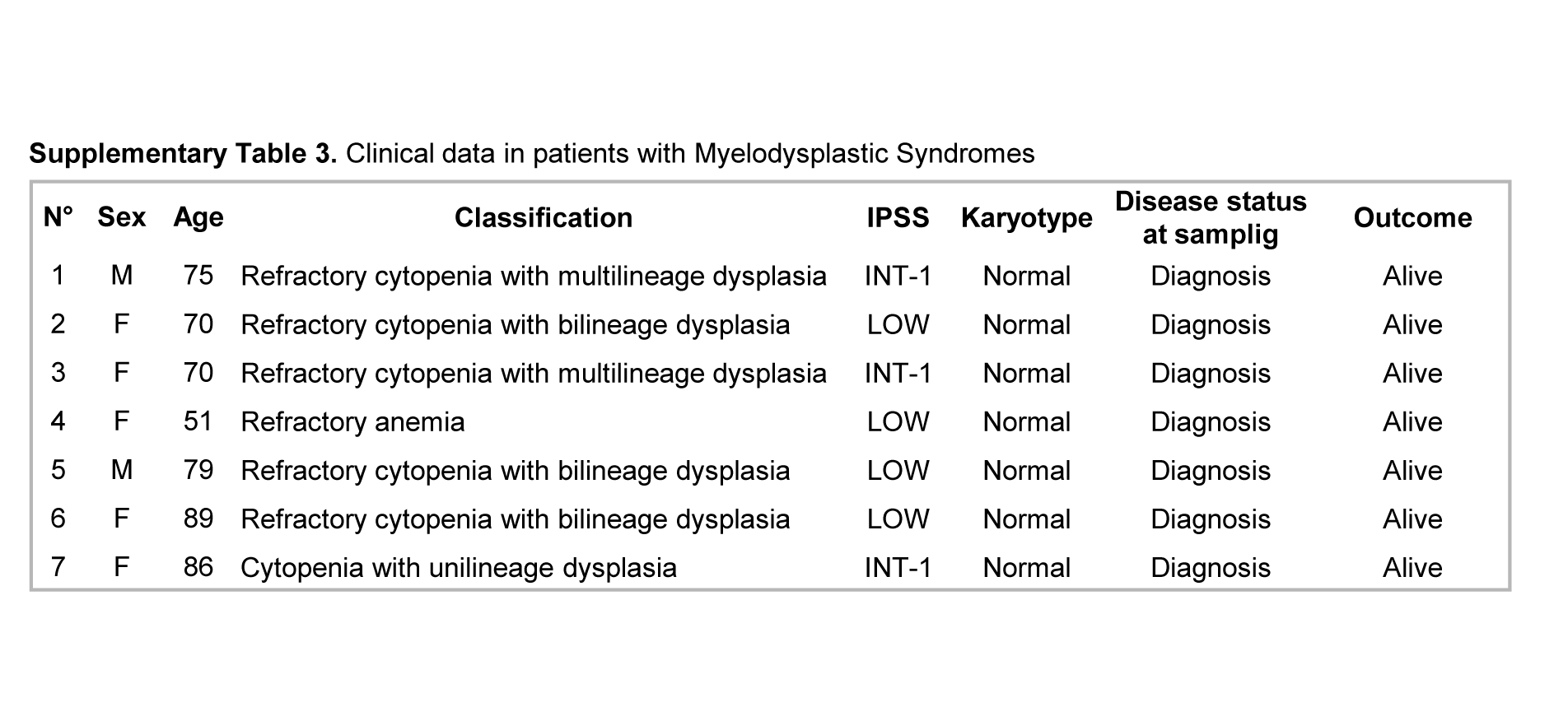

Supplement: Table S3 — Clinical data in patients with Myelodysplastic Syndromes. (TIF) [file pone.0068080.s008.tif]
