# Supplementary material for: A Novel, Non-canonical Splice Variant of the Ikaros Gene Is Aberrantly Expressed in B-cell Lymphoproliferative Disorders
Source: PLoS One. 2013 Jul 9;8(7):e68080. doi: 10.1371/journal.pone.0068080 (PMC3706598; doi:10.1371/journal.pone.0068080)
Supplement: Text S1 — Supplementary Material and Methods. (DOCX) [file pone.0068080.s009.docx]

**Text S1. Supplementary Materials and Methods**

**Cell lines**

BM, RS, YA (Allogeneic Epstein-Barr virus-transformed B cell line of a different HLA background) [Supplementary Reference 1], U937 (human leukemic monocyte lymphoma cell line), THP1 (human acute monocytic leukemia cell line), HL60 (human promyelocytic leukemia cells), Raji, BL41, Daudi (human Burkitt’s lymphoma cell lines), RPMI 8226 (human myeloma cell line) and SUDHL4 (follicular B-cell lymphoma cell line) (kindly provided by Prof. A. Faggioni) [Supplementary Reference 2] cells were cultured in RPMI 1640 with 10% heat-inactivated fetal bovine serum (FBS), 1% L-glutamine and 1% Penicillin Streptomycin. LP-1 (human myeloma cell line) (kindly provided by Prof. A. Faggioni) was cultured in in RPMI 1640 with 20% heat-inactivated fetal bovine serum (FBS), 1% L-glutamine and 1% Penicillin Streptomycin.

**Plasmids**

pcDNA3.1-Ik2 and pcDNA3.1-Ik11 were constructed by inserting the *EcoRI* fragment of pcRII-Ik2 or pcRII-Ik11 into the *EcoRI* site of pcDNA3.1 (Life Technology, Carlsbad, CA, USA). The strategy to generate pcDNA3.1-Ik6 was as follows: exon 2/3 fragment was generated by PCR (F 5'-ACTGGAATTCGCCCTTGAGGACCAT-3'; R 5'-ACTGTGGCCACGACTCTGTCACTC-3') and digested with *EcoRI/MscI*; the first 154 bp of exon 7 were amplified by PCR (F 5'-P-GGGACAAGGGCCTGTCCGACACGCCCTAC-3'; R 5'-ACTGCCCGGGGGCGTCTGCACCAGCGGG-3') and digested with *XmaI*; the remaining 563 bp of exon 7 were obtained by *XmaI/EcoRI* digestion of pcRII-Ik2. Next, the three fragments were ligated into the *EcoRI* site of pcDNA3.1. To generate pcDNA3.1/Myc-HysB-Ik11, a pcRII-Ik11 lacking the stop codon (pCRII-Ik11Δstop) was cut with *EcoRI* and inserted into the *EcoRI* site of pcDNA3.1/Myc-HysB. To generate pcDNA3.1/Myc-HysB-Ik2 and pcDNA3.1/Myc-HysB-Ik6, pcDNA3.1-Ik2 and pcDNA3.1-Ik6 were digested with *EcoRI-BstXI*. The 1198 bp *EcoRI-BstXI* Ik2 or the 768 bp *EcoRI-BstXI* Ik6 fragments were subcloned along with a 121 bp *BstX1-EcoRI* fragment of pCRII-Ik11Δstop into the *EcoRI* site of pcDNA3.1/Myc-HysB.

**SUPPLEMENTARY REFERENCES**

1. Aviner S, Yao X, Krauthgamer R, Gan Y, Goren-Arbel R, Klein T, et al. (2005) Large-scale preparation of human anti-third-party veto cytotoxic T lymphocytes depleted of graft-versus-host reactivity: a new source for graft facilitating cells in bone marrow transplantation. Hum Immunol 66:644–52.
2. Rosato P, Anastasiadou E, Garg N, Lenze D, Boccellato F, Vincenti S, Severa M, Coccia EM, Bigi R, Cirone M, Ferretti E, Campese AF, Hummel M, Frati L, Presutti C, Faggioni A, Trivedi P. (2012) [Differential regulation of miR-21 and miR-146a by Epstein-Barr virus-encoded EBNA2.](http://www.ncbi.nlm.nih.gov/pubmed/22614176) Leukemia. 26(11):2343-52.
